# Low-volume body bag cooling for severe exertional heat stroke in the emergency department: a case report

**DOI:** 10.1186/s12245-026-01321-y

**Published:** 2026-07-29

**Authors:** Takao Ikeda, Taketo Watase, Takashiro Kondo, Hiroko Watase, Tomohiro Funabiki, Mitsunaga Iwata

**Affiliations:** Department of General Internal Medicine/Emergency Medicine, Fujita Health University Hospital, 1-98 Dengakugakubo, Kutsukake-cho, Toyoake, Aichi 470-1192 Japan

**Keywords:** Exertional heat stroke, Body bag, Tarp-assisted cooling with oscillation (TACO), Cooling rate, Cold water immersion (CWI)

## Abstract

**Background:**

Exertional heat stroke (EHS) is a life-threatening emergency in which delayed or inadequate cooling substantially increases the risk of death and permanent neurological injury. Core temperature reduction to below 39 °C within 30 min is the therapeutic target, and cold-water immersion achieves the fastest cooling rates (0.15–0.35 °C/min) but is frequently unavailable in emergency departments (ED) due to equipment and space limitations. Body bag-based tarp-assisted cooling with oscillation (TACO) has been proposed as an alternative using universally available materials; however, clinical evidence supporting its emergency department use remains limited.

**Case presentation:**

A 57-year-old immunocompromised male construction worker (acute myeloid leukemia, status post umbilical cord blood transplantation, maintained on prednisolone and tacrolimus) presented with EHS following 90 min of outdoor labor in ambient conditions of 36 °C and 65% relative humidity. On ED arrival, rectal temperature was 42.0 °C, Glasgow Coma Scale score was 4, blood pressure was 125/80 mmHg, and heart rate was 183 beats/minute. Body bag-based TACO was initiated immediately upon arrival using 15 L of tap water and 15 kg of ice. Endotracheal intubation for airway protection was performed concurrently, demonstrating patient accessibility during cooling. Target temperature (rectal temperature < 39 °C) was achieved in 39 min at a cooling rate of 0.085 °C/min. Glasgow Coma Scale score improved to 14 within 7.5 h of admission. The hospital course was complicated by bacteremia requiring prolonged antimicrobial therapy. The patient was discharged ambulatory on hospital day 42 without neurological sequelae.

**Conclusions:**

Body bag-based TACO required substantially less water (15 L) than the water/ice volumes reported in TACO studies (114–151 L) using materials available in any ED. Although the cooling rate achieved (0.085 °C/min) fell below published benchmarks, the patient achieved complete neurological recovery, suggesting that immediate initiation of cooling may help offset a suboptimal rate. While a single case cannot establish equivalence to cold-water immersion, body bag-based TACO offers a practical, low-cost option for initiating cooling when standard infrastructure is unavailable, and merits further comparative evaluation.

## Background

Exertional heat stroke (EHS) represents a leading cause of sudden death among athletes and outdoor workers, with incidence increasing during summer months when ambient temperature and humidity rise [1]. The condition is characterized by core body temperature exceeding 40.5 °C with central nervous system dysfunction, rapidly progressing to multi-organ failure if left untreated [2–4]. Reported mortality rates range from 0 to 27% depending on disease severity and cooling adequacy [3, 5, 6]. Among survivors, 10–28% develop persistent cognitive and motor impairment, most commonly cerebellar dysfunction with imaging evidence of cerebellar, hippocampal, and thalamic damage, unchanged at 1- to 2-year follow-up [3]. 

Rapid cooling constitutes the definitive treatment. Guidelines identify reduction of core temperature to below 39℃ within 30 min of recognition as the therapeutic target, beyond which the risk of the mortality and neurological sequelae rises substantially [4, 7]. 

This time-dependence is closely tied to cooling rate. In the largest available case-series analysis of exertional heat stroke (521 patients), cooling rates below 0.15℃/min were associated with 4.41% mortality (23/521) and medical complications in 22.46% of survivors (117/521), versus 0% mortality and complications in only 0.77% (4/521) among patients cooled at rates exceeding this threshold—a 4.57-fold increased complication risk (95% CI, 3.42–6.28) [5]. 

Current evidence supports cold-water immersion (CWI) as the most effective modality, achieving cooling rates of 0.15–0.35℃/min [5, 7, 8]. 

However, CWI implementation in emergency departments (EDs) remains challenging due to equipment unavailability, space constraints, difficulty monitoring immersed patients or performing concurrent medical procedures [8]. 

Tarp-assisted cooling with oscillation (TACO) has emerged as a promising alternative, using waterproof material filled with ice water for whole-body cooling. Published studies report TACO cooling rates of 0.076–0.19℃/min in experimental settings [8–10]. 

The technique requires readily available materials and allows continuous monitoring and airway management. Recent case reports describe successful ED implementation using body bags—universally available equipment—as TACO delivery systems [11]. 

Despite growing evidence, body bag-based TACO implementation in EDs remains limited, and its clinical feasibility in real-world emergency settings is poorly characterized. We present a case of severe EHS successfully treated with immediate body bag-based TACO upon ED arrival, demonstrating that universally available equipment can achieve favorable neurological outcomes even when optimal cooling resources are unavailable and cooling rates are suboptimal.

## Case presentation

A 57-year-old male construction worker was brought to the ED via physician-staffed ambulance following collapse at a construction site. The patient had a history of acute myeloid leukemia status post umbilical cord blood transplantation 7 months ago and was maintained on immunosuppressive therapy (prednisolone 5 mg, tacrolimus 0.2 mg). He had returned to work the previous day without adequate heat acclimatization. While performing outdoor pipe-cutting work in ambient conditions of 36.0℃ and 65% humidity, the patient experienced dizziness after 90 min of labor and subsequently collapsed with witnessed seizure-like activity at 09:55. Emergency medical services arrived within 26 min, and prehospital cooling was initiated with cold intravenous fluids (500 mL) at 10:21. During transport, he presented with Glasgow Coma Scale score of 3 (E1V1M1), blood pressure 72/49 mmHg, heart rate 155 /minute, respiratory rate 30 /minute, and axillary temperature 42.0℃. On arrival at 10:30, rectal temperature was 42.0℃ and bladder temperature was 40.1℃, blood pressure 125/80 mmHg, and heart rate 183 beats/minute. Physical exam revealed GCS score of 4 (E1V2M1) and marked skin warmth. Initial laboratory findings showed mild hyponatremia (126 mEq/L), elevated creatine kinase (217 U/L), transaminitis (aspartate aminotransferase [AST] 244 U/L, alanine aminotransferase [ALT] 145 U/L), and elevated lactate levels (43.0 mg/dL). No coagulopathy was observed.

We initiated TACO using a body bag upon arrival. The cooling apparatus consisted of a body bag filled with 15 L of tap water and 15 kg of ice cubes, prepared in advance. Approximately half of the patient’s body was immersed. Given the profound altered mental status and risk for airway obstruction, endotracheal intubation was performed **while the patient remained immersed in the cooling apparatus**, using ketamine and rocuronium, demonstrating that airway management need not interrupt cooling. Water temperature was not measured due to the emergent situation.

**Fig. 1:**
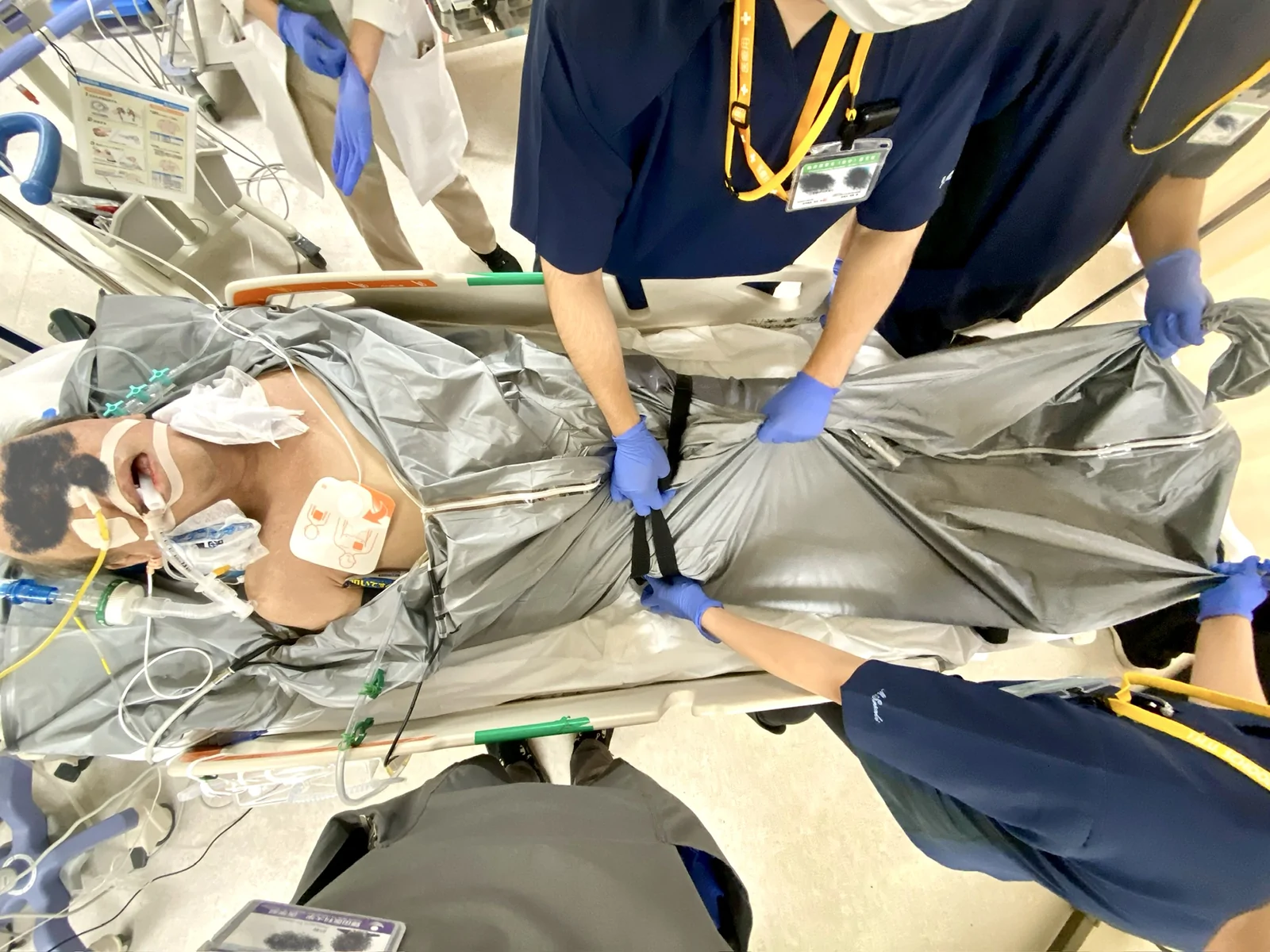
Clinical implementation of TACO. The patient is placed inside the body bag filled with 15 L of tap water and 15 kg of ice. Continuous monitoring was maintained using waterproof monitoring devices positioned on body areas above the water surface

The patient was continuously monitored via defibrillator pads (no arrhythmia), ear-lobe pulse oximetry, and intermittent right upper arm blood pressure, alongside continuous bladder and intermittent rectal temperature monitoring (Fig. 1).

A team comprising two physicians, two nurses, and three medical students implemented continuous oscillation by manually agitating the bag from four corners. Adjunctive cooling measures included ice packs applied to bilateral axillae and groins. Simultaneous resuscitation included cold intravenous of acetated Ringer’s solution (total of 2,500 mL during ED stay).

**Fig. 2:**
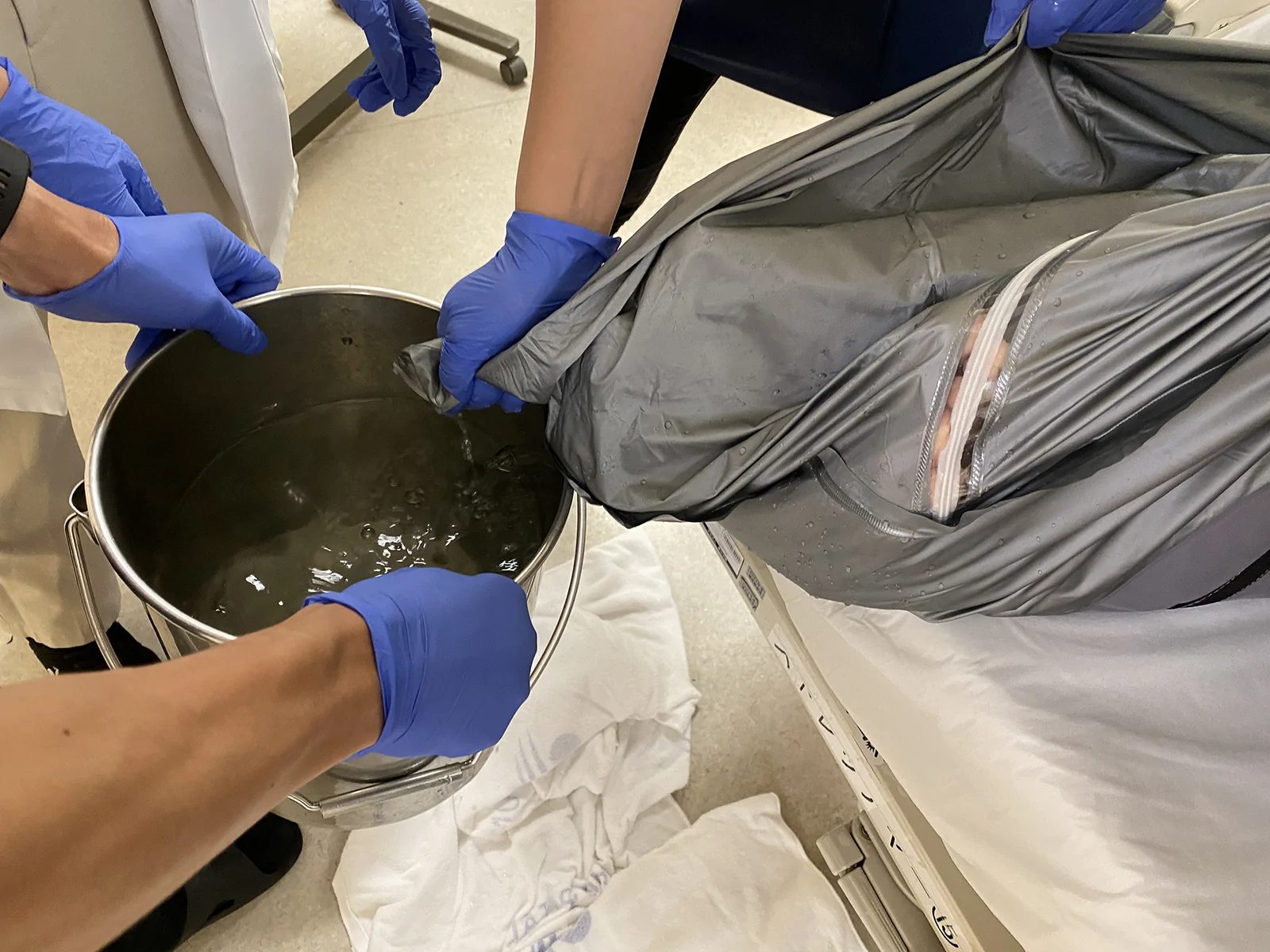
Fluid drainage from the body bag. Following achievement of target temperature, one corner of the body bag was cut to drain the ice water

TACO was discontinued at 11:09 when rectal temperature reached 38.7℃, yielding a cooling rate of 0.085℃/minute over 39 min. After reaching the target temperature, one corner of the body bag was cut to drain the water (Fig. 2).

The patient was admitted to the intensive care unit for continued monitoring and supportive care. Consciousness improved to GCS of 10T (E4VTM6) within 7.5 h of hospital arrival. Serial laboratory monitoring revealed peaking of transaminases on hospital day 1 (AST 572 U/L, ALT 358 U/L) and a peak creatine kinase of 2,165 U/L on hospital day 2 consistent with hepatic injury and rhabdomyolysis. Blood cultures grew methicillin-resistant *Staphylococcus caprae* with no source. He was transferred from the ICU on hospital day 5 following overall clinical stabilization, with ongoing antimicrobial therapy. Subsequent hospital course included continued antimicrobial treatment, immunosuppressive regimen adjustment, and inpatient rehabilitation, resulting in discharge on hospital day 42 ambulatory and without neurological sequelae, with no organ dysfunction beyond the rhabdomyolysis and hepatic injury.

## Discussion and conclusion

Although the cooling rate achieved with body bag-based TACO remained below guideline thresholds, the patient recovered without neurological sequelae —the central discrepancy discussed below in relation to existing CWI and TACO evidence. CWI remains the gold standard for EHS treatment, with cooling rates typically 0.15–0.35 °C/min [5, 7]. When implemented immediately, CWI achieves 100% survival in large case series [12, 13]. However, CWI requires specialized equipment, including immersion tubs, substantial volumes of ice water, and adequate space—not readily available in many EDs particularly in smaller or community hospitals [7, 8]. 

Controlled studies demonstrated TACO cooling rates of 0.14–0.17℃/min, exceeding the minimum acceptable threshold of 0.155℃/min for effective EHS treatment [9, 10]. Our body bag modification offers several advantages: a contained cooling environment, reduced water volume (15 L versus 114–151 L used in these prior TACO studies), minimal floor contamination, and feasibility of advance preparation [9, 10]. 

Our cooling rate (0.085 °C/min) falls below the ideal threshold (> 0.15℃/min) reported in recent guidelines [8]. Several factors may explain this suboptimal rate. First, ice water temperature was not measured and may have exceeded the < 10 °C typically used in CWI protocols. Colder water optimizes cooling through increased thermal gradient, though systematic reviews have not consistently demonstrated ice water’s superiority over cold water immersion [6]. 

Second, manual agitation of the body bag may have been less vigorous than oscillation achieved with traditional TACO using larger tarps [9]. Third, the patient’s body habitus (BMI 21.3 kg/m²) may have affected heat transfer during cooling, though the relationship between body composition and cooling rate in this modified method requires further investigation.

Despite the suboptimal cooling rate, our patient achieved complete neurological recovery. Current guidelines prioritize cooling modalities achieving ≥ 0.155 °C/min, while acknowledging that such rates may not be feasible or available in every setting, in which case the fastest practically available method should be selected [7]. The same guidelines also note that direct evidence linking time-to-target-temperature with clinical outcomes remains limited, based on small, statistically underpowered retrospective studies [7]. Our case may illustrate this latter point: cooling was initiated within minutes of arrival, and target temperature was reached within 39 min. We interpret this favorable outcome cautiously, as potentially reflecting the benefit of rapid initiation in a resource-limited setting rather than validation of this cooling rate as generally sufficient. Recent case reports of body bag cooling for heat stroke similarly demonstrate favorable outcomes [11, 14]. 

The body bag configuration may limit body surface contact compared to full immersion, potentially explaining slower cooling. Continuous core temperature monitoring becomes more challenging, though we successfully maintained both bladder and rectal measurements. The method requires adequate staffing (as noted above), which may not be universally available. Additionally, our single case limits generalizability, though published body bag cooling reports demonstrate reproducible favorable outcomes [11, 14]. 

Future research should investigate optimal parameters and conduct comparative studies against CWI and TACO to inform evidence-based guidelines for resource-limited settings.

This single case does not establish that body bag-based TACO achieves cooling rates equivalent to established EHS benchmarks, nor that a rate below these thresholds is broadly acceptable. It does demonstrate that immediate cooling initiation with universally available equipment is feasible in a resource-limited ED and was associated with a favorable neurological outcome despite a suboptimal rate. Consistent with current guidance to prioritize the fastest cooling method practically available when ideal modalities cannot be implemented [7], we suggest that body bag-assisted TACO be regarded as a time-critical bridging strategy when CWI is unavailable, rather than as an equivalent substitute. Comparative studies against standard CWI and conventional TACO are needed before this technique can be recommended for routine use in heat stroke protocols.

## Data Availability

All data generated or analysed during this study are included in this published article.

## Abbreviations

ALT: Alanine aminotransferase
AST: Aspartate aminotransferase
BMI: Body mass index
CWI: Cold-water immersion
ED: Emergency department
EHS: Exertional heat stroke
GCS: Glasgow Coma Scale
ICU: Intensive care unit
TACO: Tarp-assisted cooling with oscillation
